# Three types of alopecia in one patient: A case of central centrifugal cicatricial alopecia, alopecia areata, and telogen effluvium

**DOI:** 10.1016/j.jdcr.2025.06.028

**Published:** 2025-06-30

**Authors:** Shanelle Jackson, Charissa N. Obeng-Nyarko, Kevin Puerta Durango, John T. Seykora, Susan C. Taylor

**Affiliations:** a Michigan State University College of Human Medicine, Grand Rapids, Michigan; b Florida State University College of Medicine, Tallahassee, Florida; c Geisel School of Medicine at Dartmouth, Hanover, New Hampshire; d Department of Dermatology, Perelman School of Medicine at the University of Pennsylvania, Philadelphia, Pennsylvania

**Keywords:** alopecia areata, central centrifugal cicatricial alopecia, complex alopecia, female pattern hair loss, frontal fibrosing alopecia, lichen planopilaris, telogen effluvium, traction alopecia

## Introduction

There are diverse types of alopecia, and patients often do not fit under a single diagnostic label. Patients may present with multiple overlapping forms of alopecia, complicating diagnosis and management. Notably, persons of African heritage frequently demonstrate a pattern of multiple concurrent forms of hair loss, often involving both scarring and nonscarring types.^1^ Genetic, inflammatory, mechanical, and environmental factors can influence patterns of hair loss. Underdiagnosis and misdiagnosis in patients with skin of color (SOC) can lead to delayed treatments and poorer outcomes. Although instances of concurrent alopecia areata (AA) and central centrifugal cicatricial alopecia (CCCA) have been documented, their infrequency in the literature may be attributed to the diagnostic difficulties associated with these conditions.^2^

Hair loss can be psychologically distressing, affecting mental health, self-esteem, and social interactions, and leading to a significant negative impact on quality of life.^3^ Early and precise diagnosis of all contributing factors is critical to minimizing emotional distress, promoting patient well-being, increasing management effectiveness, and improving patient outcomes.

In this report, we present a patient with 3 distinct types of alopecia: AA, which is characterized as an autoimmune nonscarring alopecia; CCCA, as a chronic scarring alopecia; and telogen effluvium, characterized as a reactive nonscarring hair loss. This case underscores the need for a thorough clinical assessment to differentiate and manage coexisting conditions and increases awareness of complex alopecia, especially in patients with SOC.

## Case report

**Fig 1:**
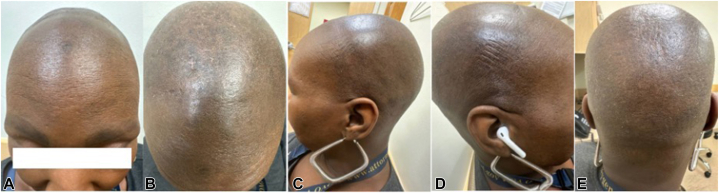
Diffuse hair loss **(A)** frontal, **(B)** parietal, **(C)** right frontotemporal, **(D)** left frontotemporal, and **(E)** occiput scalp photography.

A 48-year-old African American woman presented to the dermatology office with a 6-month history of marked diffuse hair loss on the scalp. Her past surgical history was significant for gastric bypass, resulting in a weight loss of over 100 pounds within 18 months, and her past medical history was notable for deficiencies in vitamin B12, vitamin D, and iron. Her family history was notable for her mother having hair loss, but the type of alopecia was unknown. On physical examination, the patient exhibited loss of eyebrows bilaterally and diffuse hair loss (Fig 1). No erythema, scale, crust, or pus was identified. Trichoscopy examination revealed diffuse alopecia with a few short-broken hairs in the occiput. Areas of hyperpigmentation and lichenification were also noted in the occiput. The patient reported 3/10 pruritus and 2/10 flaking of the scalp but denied pain, tenderness, burning, or soreness of the scalp. A scalp biopsy of the vertex and mid-scalp was performed.

**Fig 2:**
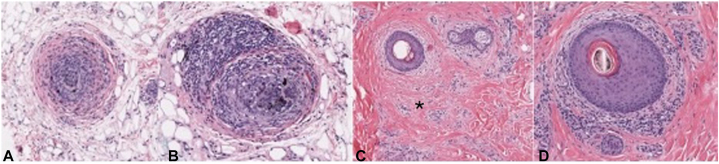
Mid-scalp specimen. **A** and **B,** Hair bulbs in the subcutaneous fat demonstrate a prominent peribulbar and intrabulbar mononuclear cell infiltrate comprised of mature lymphocytes. Scattered melanophages were noted. **C,** The image demonstrates focal follicular scarring associated with mild perifollicular fibrosis. **D,** This image shows prominent perifollicular lymphocytic inflammation associated with fibroplasia and thinning of the outer root sheath. ∗, Perifollicular fibrosis.

**Fig 3:**
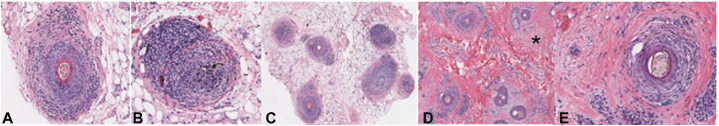
Vertex specimen. **A,** Transverse section of a hair follicle associated with a prominent perifollicular inflammatory infiltrate primarily comprised of lymphocytes. **B,** Hair bulb associated with a prominent lymphocytic infiltrate and subcutaneous fat. **C,** Cross-sections of a hair follicle and hair bulb associated with prominent lymphocytic inflammation. **D,** Lower magnification view of a horizontal section demonstrating focal follicular scarring. **E,** Horizontal section of a hair follicle associated with perifollicular lymphocytic inflammation and mucinous fibroplasia. Thinning of the outer root sheath was prominent. ∗, Focal follicular scarring.

The histological findings from a biopsy of the vertex and mid-scalp scalp were notable for increased miniaturized follicles, scattered follicular scars, perifollicular lymphocytic inflammation, vacuolar change, scattered necrotic keratinocytes, a focally intense lymphocytic infiltrate, pigment incontinence, and occasional eosinophils. These features describe complex alopecia with scarring and features of AA in the mid-scalp (Fig 2) and scarring alopecia with focal lichenoid inflammation and increased miniaturized follicles in the vertex (Fig 3). The clinicopathologic findings included nonscarring and scarring alopecia components, with CCCA, AA, and TE all favored. Although lichen planopilaris (LPP)/frontal fibrosing alopecia (FFA) could not be ruled out completely, the clinical findings of hair loss in the crown were less supportive of this diagnosis. Additionally, minimal lichenoid inflammation did not support LPP or FFA on histology.

Based on clinical and pathologic findings, the official diagnosis was concluded to include CCCA, AA, and TE. Our patient was started on doxycycline monohydrate 100 MG capsules twice daily, clobetasol 0.05% cream daily, triamcinolone 5 ng/dL scalp injections every 2 months, and tofacitinib 2% cream twice daily. After starting this treatment plan, our patient was lost to follow-up.

## Discussion

There are 2 primary classes of alopecia: scarring and nonscarring. Scarring alopecias include CCCA, LPP, FFA, and chronic traction alopecia. Non-scarring alopecias include AA, TE, female pattern hair loss, and acute traction alopecia. Coexisting alopecias are important to recognize for accurate diagnosis. This case report adds to the existing literature on the simultaneous occurrence of two or more alopecias, encompassing both scarring and nonscarring types. Research has demonstrated evidence of coexisting alopecias.^1^^,^^4^ Specifically, there are studies that have highlighted the coexistence of CCCA and AA.^2^

The cause of the nonscarring component of her hair loss is multifactorial and includes both AA and TE. The biopsy results identified peribulbar lymphocytic infiltration (“swarm of bees”), melanophages, and pigment incontinence, which indicated AA. AA and TE are both likely triggered by stress in this patient. We suspect TE to be a result of the physiologic stress she endured from her history of bariatric surgery, massive weight loss, iron deficiency anemia, and vitamin deficiencies.^5^^,^^6^ TE can unmask underlying hair loss by causing widespread shedding and making it more clinically apparent.

The scarring component of her hair loss included CCCA. This was supported by her biopsy results showing follicular scaring, fibroplasia, and thinning of the outer root sheath. The inflammatory infiltrates supported an active scarring process. It is important to remain vigilant when evaluating hair loss, especially in patient populations with a higher prevalence of CCCA. The best practices for diagnosis include detailed history-gathering (eg, asking about triggers such as anemia, vitamin deficiencies, past surgeries, or stress), noninvasive tools (eg, trichoscopy to assess follicular patterns), and invasive tools (eg, scalp biopsy for definitive histopathological diagnosis). It is important to use evidence-based guidelines for treating hair loss in patients with SOC.^3^^,^^7^

The complexity of diagnosing and treating patients with multiple types of alopecia is underscored by this case. A high index of suspicion should be maintained for coexisting hair loss disorders. A multimodal approach incorporating anti-inflammatory, immunosuppressive, and anti-fibrotic treatments is essential for optimizing patient outcomes.

Tofacitinib, a JAK 1/3 inhibitor, is safe and effective in AA treatment.^8^ This substantiates our selection of tofacitinib 2% cream to target both the AA and CCCA seen in our patient. Through attention to the intricacies of complex alopecias, physicians can adequately tailor their treatment plans to each patient, as seen in our case.

## Conflicts of interest

None disclosed.
